# Endothelial cell-derived MMP19 promotes pulmonary fibrosis by inducing E(nd)MT and monocyte infiltration

**DOI:** 10.1186/s12964-023-01040-4

**Published:** 2023-03-13

**Authors:** Weiming Zhao, Lan Wang, Juntang Yang, Xinyu Chen, Xiaoshu Guo, Kai Xu, Ningdan Wang, Wenyu Zhao, Cong Xia, Hui Lian, Ivan Rosas, Guoying Yu

**Affiliations:** 1 State Key Laboratory Cell Differentiation and Regulation, Henan International Joint Laboratory of Pulmonary Fibrosis, Henan Center for Outstanding Overseas Scientists of Pulmonary Fibrosis, College of Life Science, Institute of Biomedical Science, Henan Normal University, Xinxiang, Henan China; 2 Division of Pulmonary, Critical Care and Sleep Medicine, Baylor College of Medicine, Houston, TX 77030 USA

**Keywords:** MMP19, E(nd)MT, ET1, SDF1/CXCR4, Pulmonary fibrosis

## Abstract

**Background:**

Matrix metalloproteinases (MMPs) play important roles in remodeling the extracellular matrix and in the pathogenesis of idiopathic pulmonary fibrosis (IPF). MMP19, which is an MMP, was significantly upregulated in hyperplastic alveolar epithelial cells in IPF lung tissues and promoted epithelial-mesenchymal transition (EMT). Recent studies have demonstrated that endothelial-to-mesenchymal transition (E(nd)MT) contributes to pulmonary fibrosis. However, the role of MMP19 in pulmonary vascular injury and repair and E(nd)MT remains unclear.

**Methods:**

To determine the role of MMP19 in E(nd)MT and pulmonary fibrosis. MMP19 expressions were determined in the lung endothelial cells of IPF patients and bleomycin (BLM)-induced mice. The roles of MMP19 in E(nd)MT and endothelial barrier permeability were studied in the MMP19 cDNA-transfected primary human pulmonary microvascular endothelial cells (HPMECs) and MMP19 adenoassociated virus (MMP19-AAV)-infected mice. The regulatory mechanism of MMP19 in pulmonary fibrosis was elucidated by blocking its interacting proteins SDF1 and ET1 with AMD3100 and Bosentan, respectively.

**Results:**

In this study, we found that MMP19 expression was significantly increased in the lung endothelial cells of IPF patients and BLM-induced mice compared to the control groups. MMP19 promoted E(nd)MT and the migration and permeability of HPMECs in vitro, stimulated monocyte infiltration into the alveolus, and aggravated BLM-induced pulmonary fibrosis in vivo. SDF1 and Endothelin-1 (ET1) were physically associated with MMP19 in HPMECs and colocalized with MMP19 in endothelial cells in IPF patient lung tissues. AMD3100 and bosentan alleviated the fibrosis induced by MMP19 in the BLM mouse model.

**Conclusion:**

MMP19 promoted E(nd)MT by interacting with ET1 and stimulated monocyte infiltration into lung tissues via the SDF1/CXCR4 axis, thus aggravating BLM-induced pulmonary fibrosis. Vascular integrity regulated by MMP19 could be a promising therapeutic target for suppressing pulmonary fibrosis.

**Video abstract**

**Supplementary Information:**

The online version contains supplementary material available at 10.1186/s12964-023-01040-4.

## Introduction

IPF is a chronic, progressive interstitial lung disease with a low median survival rate after diagnosis [1]. It is believed that IPF is induced by several factors, including genetic factors, epigenetic factors, aging, environmental factors and infections, which culminate in an uncontrolled repair process in the injured lung [2]. However, myofibroblast activation and excessive ECM deposition are recognized as pathological features of end-stage IPF [3].

MMPs are believed to be antifibrotic genes due to their ECM degradation functions [4]. Mmp13^−/−^ mice exhibited more extensive inflammation at 7 days after bleomycin (BLM) treatment, and more severe ECM deposition and prolonged lung fibrosis than wild-type (WT) mice [5]. Epithelial Mmp14 deficiency in mice also increased the severity and extensiveness of fibrotic injury and affected the resolution of the lesions [6]. However, overexpression of MMP3 in the rat lung resulted in the accumulation of myofibroblasts and pulmonary fibrosis [7]. These studies indicated that the roles of MMPs in pulmonary fibrosis are complex and multifaceted.

Our previous studies revealed that MMP19 was significantly upregulated in hyperplastic alveolar epithelial cells in IPF lung tissues, and was required for normal epithelial cell wound healing in vitro [8]. Mmp19^−/−^ lung fibroblasts showed a significant increase in proliferation, migration, and fibroblast-myofibroblast transition (FMT) [9]. Mmp19^−/−^ mice exhibited much more severe pulmonary fibrosis after BLM instillation than WT mice [8]. Studies have indicated that endothelial cells could represent another potential source of fibroblasts/myofibroblasts via E(nd)MT [10, 11], which contributes to the progression of pulmonary fibrosis [12–14]. However, MMP19 has not been studied in endothelial cells. In this study, we found that MMP19 expression was significantly upregulated in endothelial cells in IPF and BLM-induced fibrotic mouse lung tissues compared to the corresponding control groups. Thus, we hypothesized that endothelial cell-derived MMP19 promoted E(nd)MT, which led to the excessive accumulation of myofibroblasts in lung tissues and aggravated BLM-induced pulmonary fibrosis. In this study, we revealed the contribution of MMP19 in regulating E(nd)MT, monocyte infiltration and the pathogenesis of pulmonary fibrosis.

## Materials and methods

### Cell culture

Human Pulmonary Microvascular Endothelial Cells (HPMEC cells) were purchased from ScienCell (San Diego, California, Cat. No. 3000) and propagated in ECM (San Diego, California) under standard culture conditions. THP-1 cells were purchased from Procell (Wuhan, China, Cat. No. CL-0233) and propagated in RPMI-1640 (Procell, Cat. No. PM150110) under standard culture conditions.

### Animal experiment

8-week-old female C57BL/6 N mice were purchased from Vital River Laboratories (Beijing, China). Mice were narcotized by inhaling 40% isoflurane (RWD, Shenzhen, China) (diluted with 1,2-Propanediol (Aladdin, Shanghai, China)), and then were given 50 µL MMP19WT-AAV or MMP19E213A-AAV (5 × 10^12^ V·g/mL) (HANBIO, Shanghai, China) by intratracheal injection. Because recombinant adeno-associated viruses (rAAV) are promising gene transfer vectors that produce long-term expression without toxicity [15], therefore, the overexpression of MMP19 in mice lungs were achieved after AAV injection. On day 4, MMP19WT-AAV and MMP19E213A-AAV-infected mice were selected randomly and narcotized as before and intratracheal injected with 50 µL PBS or BLM (1.5 U/kg). Finally, mice were euthanatized with 20% Ethyl Carbamate (800 mg/kg, i.p) (Sigma-Aldrich, Missouri, USA) and sacrificed on day 18. For BOS (MCE, New Jersey, USA) and AMD3100 (MCE) treatment, 100 µL of BOS (100 mg/kg, gastric infusion) or 200 µL of AMD3100 (5 mg/kg, i.p) were given to mice between day 1 and day 13 after BLM instillation. The transfection efficiency of AAV was detected by In Vivo Imaging System (Berthold LB 985, Germany) on day 4, and by fluorescence microscope and western blot on day 18. Bronchoalveolar lavage (BAL) fluid was collected via trachea insertion. Lung hydroxyproline content was analyzed using a Hydroxyproline Assay Kit (Sigma-Aldrich). Data are expressed as µg hydroxyproline/g lung. No blinding method was used. All animal handling procedures were performed following protocols approved by the Institutional Care and Ethics Committee of Henan Normal University (No. 2018-11-15) and conforming to the NIH guidelines (Guide for the Care and Use of Laboratory Animals).

### Cell transfection and treatment

MMP19WT and MMP19E213A (a catalytically inactive mutant) cDNA with vector pCMV-sport6 (Open Biosystems, Lafayette, CO) were transfected into HPMEC cells with Lipofectamine^®^ 3000 (Invitrogen, Carlsbad, USA) following the instructions. For BOS treatment, HPMEC cells were pre-treated with 10 µM BOS for 24 h before MMP19WT and MMP19E213A cDNA were transfected. For recombinant human MMP19 (rhMMP19) treatment, HPMECs were treated with 500 ng/mL rhMMP19 and incubated for 24 h.

### Cell migration and scratch assay

Transwell (Corning, NewYork, USA) was used to perform the migration assays. The HPMECs transfected with MMP19WT and MMP19E213A cDNA were labeled with 1,1′-dioctadecyl-3,3,3,3′-tetramethylindocarbocyanine perchlorate (DiI) and seeded into the transwell inserts with serum-free medium and 10% FBS medium at the bottom. After 24 h, the number of cells that penetrated the pores was counted using an inverted fluorescence microscope (Nikon, Tokyo, Japan).

HPMEC were cultured on a 6-well plate and transfected with MMP19WT and MMP19E213A cDNA and growing to 100% confluence. In a sterile environment, a 200 µL pipette tip was used to make a vertical wound down through the cell monolayer, take a snapshot picture and check for wound closure at 0, 24 and 48 h with an inverted microscope.

### Cell monolayer permeability

The HPMECs transfected with MMP19WT and MMP19E213A cDNA were growing as monolayers on fibronectin-coated transwell plates for 48 h. FITC-albumin (5 mg/mL) was added to the upper chamber of the transwell, and was allowed to equilibrate for 1 h. Culture media (200 µL) collected from the lower chambers were analyzed for FITC fluorescent intensity using a fluorometric plate reader at excitation 494 nm.

### Immunocytochemistry

The HPMECs transfected with MMP19WT and MMP19E213A cDNA were fixed with 4% paraformaldehyde (10 min), and then permeabilized with 0.1% Triton X-100 (5 min), followed by blocking with 10% goat serum (30 min), cells were incubated with anti-CD31 antibody (1:100; Abcam, Cambridge, UK, ab76533), anti-VE-Cadherin (1:100; Abcam, ab33168) and anti-α-SMA antibody (1:100; Abcam, ab240654) overnight at 4 °C, followed by incubation for 1 h at RT with Goat Anti-Mouse IgG H&L (Alexa Fluor^®^ 488) (1:1000; Abcam, ab150113) and Goat Anti-Rabbit IgG H&L (Alexa^®^ Fluor 647) (1:1000; Abcam, ab150079). Cell nuclei were labeled with DAPI. Images were obtained with a Zeiss microscope.

### qRT-PCR

Total RNA was isolated from the HPMECs or mice lungs by using a miRNeasy Mini Kit (QIAGEN, Dusseldorf, Germany), and performed reverse transcription using random primers and a reverse transcription kit (Promega, Madison, USA). The PCR was performed by using qPCR Master Mix (Promega) according to the manufacturer’s instructions. β-actin was used as an internal reference. Each sample was performed in triplicates.

### Western blot

Total protein from the mice lung tissues or cultured cells was extracted using RIPA lysis buffer that contains PMSF (Beyotime, Shanghai China). Quantification of the protein was determined by using a BCA Protein Assay Kit (Solarbio Life Sciences, Beiijing China). Total proteins (20 µg) were separated by SDS-PAGE gel and then transferred onto an Immobilon-PSQ PVDF Membrane (Millipore, Massachusetts, USA). After blocking with 5% (w/v) fat-free milk for 1 h, the membranes were incubated with anti-CD31 antibody (1:100; Abcam, ab281583), anti-VE-Cadherin (1:100; Abcam, ab33168), anti-α-SMA antibody (1:100; Abcam, ab240654), anti-Collagen I (1:100; Abcam, ab138492), anti-VIM (1:100; Abcam, ab20346), anti-MMP19 (1:100; Affinity, AF0215), anti-N-cadherin (1:100; Abcam, ab76011), anti-SDF1 (1:100; Affinity, AF5279) and anti-ET1 (1:100; Abcam, ab2786) overnight at 4 °C. The corresponding horseradish-peroxidase-conjugated (HRP) Goat Anti-Mouse IgG (Abcam, ab6789) or Goat Anti-Rabbit IgG (Abcam, ab6721) (1:50000; Abcam) was applied for 1 h at room temperature. Immunoreactive bands were rinsed with enhanced chemiluminescence reagents (Millipore) and visualized in a Molecular Imager ChemiDoc XRS System (Bio-Rad, USA). β-actin was used as an internal reference.

### Measurement of pulmonary endothelial barrier permeability

Thirty minutes before the mice were sacrificed, a solution of 2% Evans Blue (EB) (20 mg/kg, Sigma Aldrich) in 0.9% saline was injected via the femoral vein and left to circulation. Then the lungs were flushed to remove blood (EB containing) from vasculature and mice were sacrificed and whole lungs were harvested. Dry the tissues in a drying oven at 60 °C, on foil, for 48 h. The lungs for EB extraction were homogenized in trichloroacetic acid (1 mL/100 mg tissues) and kept at room temperature for 24 h. Then the homogenates were centrifuged (at 10,000×g for 12 min at 4 °C), and the supernatant was collected and diluted with 1:3 volumes of 95% ethanol before photospectrometric determination of EB concentration (fluorescence: excitation at 590 nm, emission at 645 nm, absorbance at 620 nm). Results were normalized to the tissue weight.

### Immunofluorescence

Mice or IPF paraffin-embedded tissue sections were deparaffinized in xylene, 95, 90, 80 and 70% ethanol successively, followed by PBS. Sections were permeabilized with 0.3% Triton X-100 and co-immunostained with anti-CD31 (1:100; Thermo, Waltham, USA, MA1-26196), anti-α-SMA (1:100; Abcam, ab150301), anti-MMP19 (1:100; Affinity, AF0215), anti-ET1 (1:100; Abcam, ab2786), or anti-SDF1 (1:100; Santa Cruz Biotechnology, sc-74,271) overnight at 4 °C, followed by incubation with Goat Anti-Mouse IgG H&L (Cy5) (1:1000; Servicebio, Wuhan, China, GB27301), Goat Anti-Mouse IgG H&L (Alexa Fluor^®^ 488) (1:1000; Abcam, ab150113), Goat Anti-Rabbit IgG H&L (Alexa Fluor^®^ 647) (1:1000; Abcam, ab150079), Goat Anti-Mouse IgG H&L (Alexa Fluor^®^ 647) (1:1000; Abcam, ab150115), or Goat Anti-Rabbit IgG H&L (Alexa Fluor^®^ 488) (1:1000; Abcam, ab150077) for 1 h at RT. Cell nuclei were labeled with DAPI. Images were obtained with a Zeiss microscope or a confocal laser scanning microscope (Leica, WETZLAR, Germay). Lung samples from IPF patients were collected in Xinxiang central hospital with informed consent. The study was approved by the Xinxiang central hospital Medical Research Ethics Committee (No.2019-01-12). The research conformed to the principles of the Helsinki Declaration.

### Immunohistochemistry

Mice or IPF paraffin-embedded tissue sections were deparaffinized and then permeabilized with 0.3% Triton X-100 (5 min), followed by blocking with endogenous peroxidase blocking buffer (10 min) (Beyotime), antigen retrieval was carried out by heat mediation in a citrate buffer solution (pH = 6) (20 min). Sections were closed in a blocking buffer (60 min) (Beyotime) and immunostained with anti-α-SMA (1:100; Abcam, ab240654), anti-MMP19 (1:100; Affinity, AF0215), anti-ET1 (1:100; Abcam, ab2786), anti-SDF1 (1:100; Affinity, AF5279), anti-collagen I (1:100; Abcam, ab138492), anti-collagen IV (1:100; Abcam, ab6586), anti-CXCR4 (1:100; Abcam, ab181020), anti-EMR1 (1:100; Affinity, DF2789) overnight at 4 °C, followed by incubation for 1 h at RT with biotin-conjugated goat anti-mouse or goat anti-rabbit secondary antibodies followed by incubating with SABC (1:100; Beyotime), and then visualized by DAB stain (Beyotime). Cell nuclei were labeled with hematoxylin.

### Co-Immunoprecipitation

Co-IP was performed using a Pierce™ Classic Magnetic IP/Co-IP Kit (Thermo Scientific, Massachusetts, USA) according to the manufacturer’s instructions. Briefly, HPMECs were washed carefully with PBS after discarding the culture medium. Ice-cold IP Lysis/Wash Buffer was added and then incubated for 30 min on ice with periodic mixing. Transfer supernatant to a new tube for protein concentration determination after concentrating at ~ 13,000 ×g for 10 min to pellet the cell debris. Combine cell lysate with 8 µg of anti-MMP19 (Thermo, PA124424), anti-ET1 (Abcam, ab2786) and anti-SDF1 (CST, Danvers, USA, 3530) with 800 µg sample. Incubate at 4 °C overnight to form the immune complex. Add 25 µL (0.25  mg) of Pierce Protein A/G Magnetic Beads to antigen sample/antibody mixture to the tube containing pre-washed magnetic beads and incubate at room temperature for 3 h with mixing. Collect the beads with a magnetic stand and remove the unbound sample and save them for analysis.

### Monocyte adhesion assay

THP-1 cells were pre-treated with 10 ug/mL AMD3100 (MCE) for 24 h, and labeled with DiI for 10 min. The labeled THP-1 cells were seeded at a density of 5.0 × 10^5^ cells/mL on confluent HPMECs, which were transfected with MMP19 cDNA 24 h ago. Following 1 h of incubation, non-adherent cells were removed by gentle washing with PBS and THP-1 cell adhesion was assessed using an inverted fluorescence microscope (Nikon).

### Statistical analysis

Data were analyzed by Student *t* tests and ANOVA. Statistical analyses were performed using GraphPad Prism version 7.0 (GraphPad Software, Inc. Serial: GPS-0320559-LFUL-95,242). Statistical tests are justified as appropriate for every figure, data are presented as mean ± SD and were considered statistically significant at *P* less than 0.05. Differences between two groups were assessed with the two-tailed Student’s unpaired t test. The one-way ANOVA with Tukey’s multiple comparison test was used to assess differences between more than two groups. No statistical methods were used to predetermine the sample size. Mice were randomly allocated to experimental groups. No blinding method was used for injection. There was no animal exclusion criteria. The variance was similar between the groups that were being statistically compared.

## Results

### MMP19 expression is upregulated in the endothelial cells of IPF and BLM-induced fibrotic mouse lungs

**Fig. 1:**
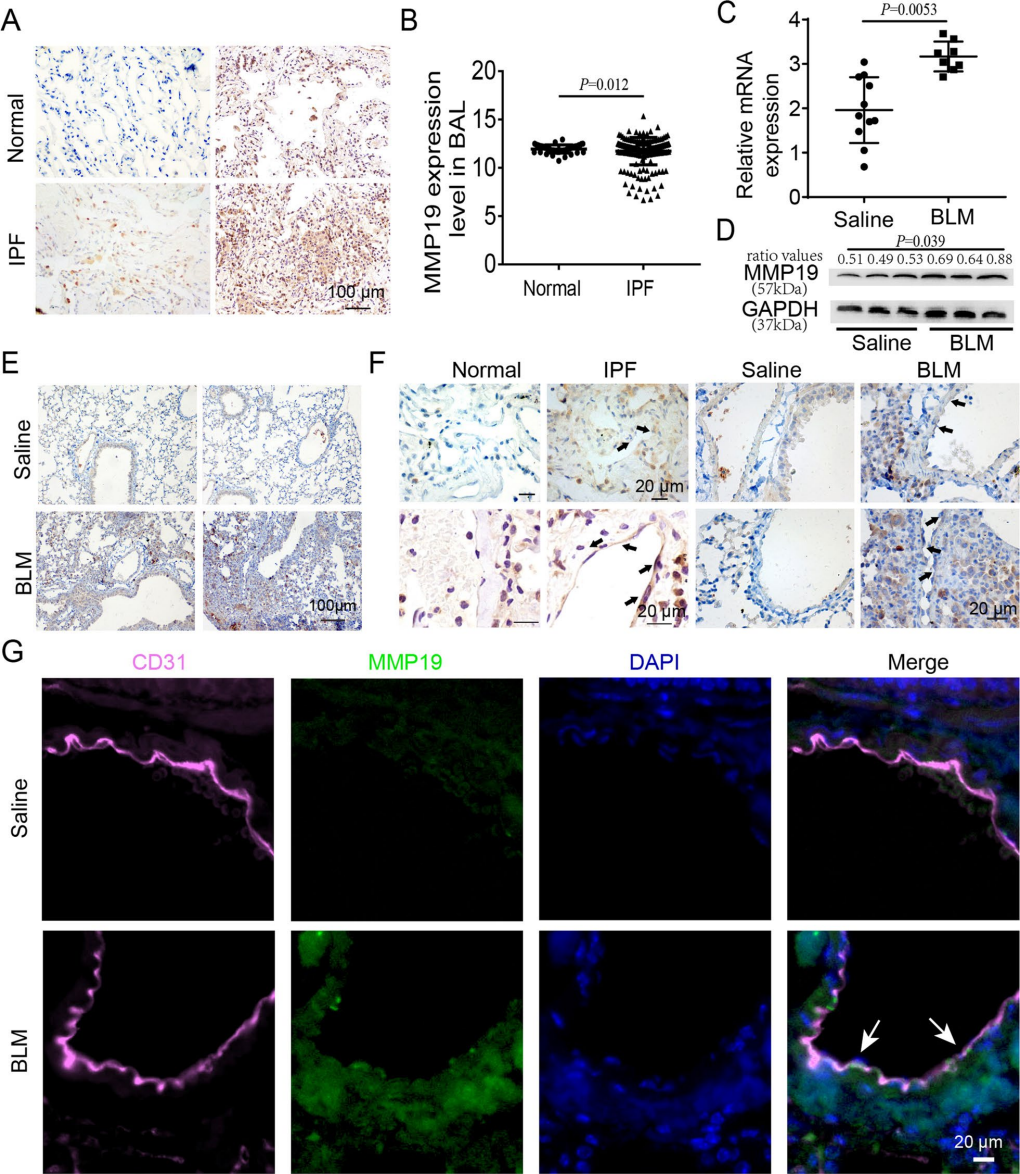
MMP19 is significantly upregulated in the endothelial cells of IPF patients and BLM-induced fibrotic mouse lungs. **A** Immunohistochemistry analysis for MMP19 in IPF lung tissue. **B** MMP19 expression level in lung BAL fluid of IPF and normal control. **C** qRT-PCR was performed for the expression of MMP19 in lung homogenates from mice treated with saline or BLM. **D** Immunoblot analysis of MMP19 in lung homogenates from mice treated with saline or BLM. **E** Immunohistochemical evaluation of MMP19 in saline control or BLM-challenged mice lung. **F** Immunohistochemistry analysis for MMP19 in endothelial cells of IPF and BLM-challenged mice lung sections. **G** Immunofluorescence analysis for CD31 (pink) and MMP19 (green) in endothelial cells in mice treated with saline control or BLM

The IHC results showed intense cytoplasmic immunostaining of MMP19 protein in IPF lung tissues (Fig. 1A). GEO datasets (GSE70867) showed that MMP19 expression in the BAL fliud of IPF was significantly upregulated compared with that of normal controls (Fig. 1B). Furthermore, we measured Mmp19 expression in the mouse lung tissues by qRT-PCR, western blot and IHC analysis and found that Mmp19 expression was significantly upregulated in BLM-induced fibrotic mouse lungs compared to the control mouse lungs (Fig. 1C–E). Additionally, we examined the protein expression of MMP19 in endothelial cells by IHC analysis, and observed intense cytoplasmic immunostaining in lung endothelial cells of IPF and BLM-induced fibrotic mice, while weak staining in corresponding control lungs (Fig. 1F). In addition, confocal analysis showed that Mmp19 colocalized with CD31, and Mmp19 positive staining was observed in the endothelial cells of BLM-induced fibrotic mouse lungs, whereas staining was not observed in the normal control lungs (Fig. 1G).

### The overexpression of MMP19 aggravates BLM-induced pulmonary fibrosis

**Fig. 2:**
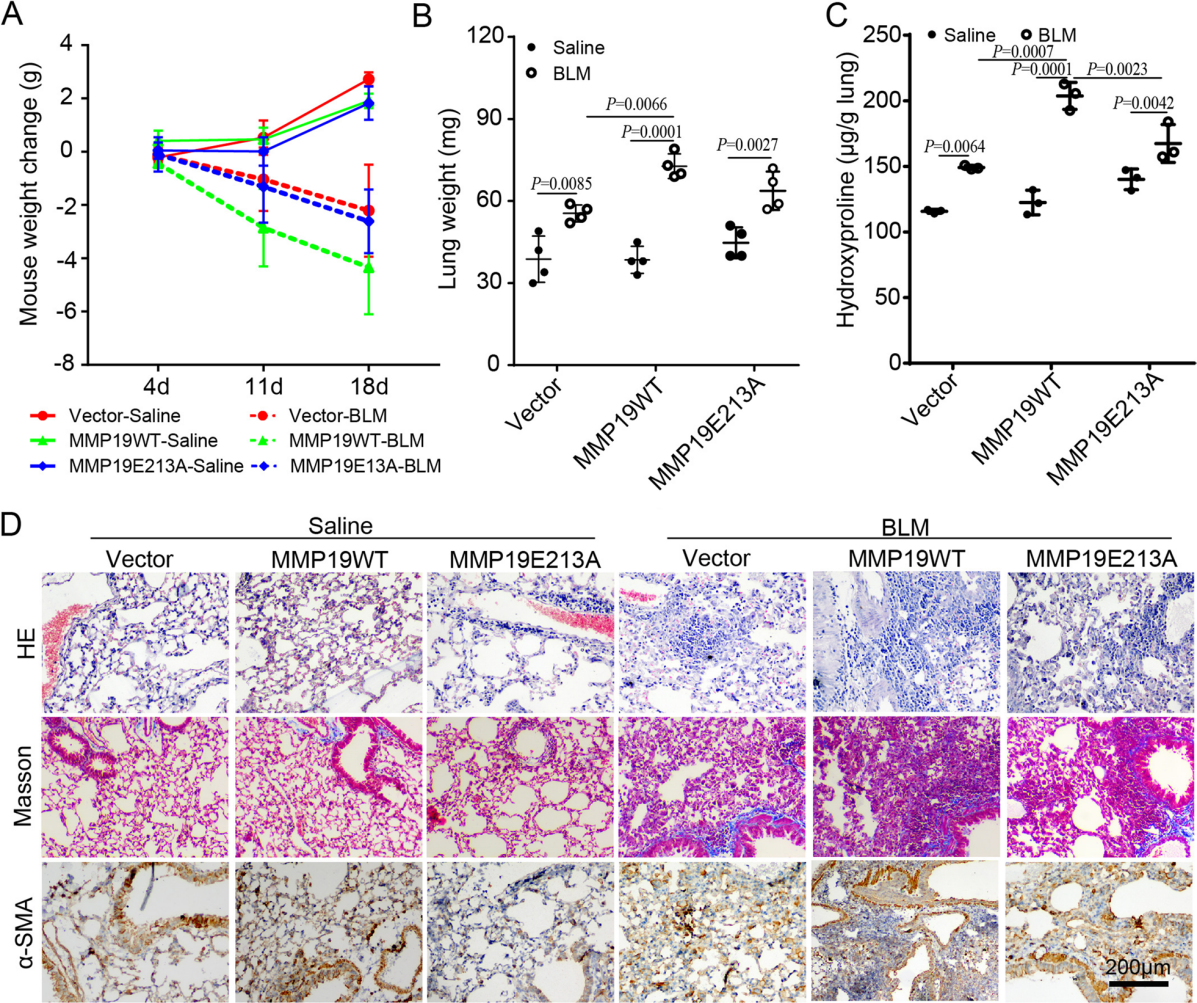
The overexpression of MMP19 aggravates BLM-induced mouse lung fibrosis. **A** Weight changes of MMP19WT- and MMP19E213A-AAV-infected mice treated with saline control or BLM. n = 4 in all groups. **B** Dry lung weight of MMP19WT- and MMP19E213A-AAV-infected mice treated with saline control or BLM. n = 4 in all groups. **C** Lung hydroxyproline levels in MMP19WT- and MMP19E213A-AAV-infected mice treated with saline control or BLM. n = 3 in all groups. **D** Representative micrographs of H&E staining, Masson’s trichrome and immunohistochemical staining of lung sections for ɑ-SMA

To determine the role of MMP19 in pulmonary fibrosis, we generated mice that overexpressed MMP19 in the lungs by intratracheal injection of AAV (Additional file 1: Fig. S1A) and evaluated the response to BLM in MMP19WT- and MMP19E213A-AAV-infected mice. An in vivo Imaging System (Additional file 1: Fig. S1B), fluorescence microscopy (Additional file 1: Fig. S1C) and western blot (Additional file 1: Fig. S1D) indicated that MMP19 was overexpressed in the lung tissues of MMP19WT- and MMP19E213A-AAV-infected mice. In addition, MMP19WT- and MMP19E213A-AAV-infected mice exhibited a significant weight loss (Fig. 2A) and increased dry lung weight (Fig. 2B) and hydroxyproline levels in lung tissue (Fig. 2C) after BLM instillation compared to those in the control group. HE and Masson trichrome staining showed that MMP19WT- and MMP19E213A-AAV-infected mice developed more severe fibrosis after BLM instillation than the control mice (Fig. 2D). Furthermore, the expression of α-SMA in MMP19WT- and MMP19E213A-AAV-infected mouse lungs after BLM instillation were increased compared with the control mice (Fig. 2D). Taken together, these results suggested that MMP19 aggravated BLM-induced pulmonary fibrosis.

### MMP19 promotes E(nd)MT, barrier permeability and migration in HPMECs

**Fig. 3:**
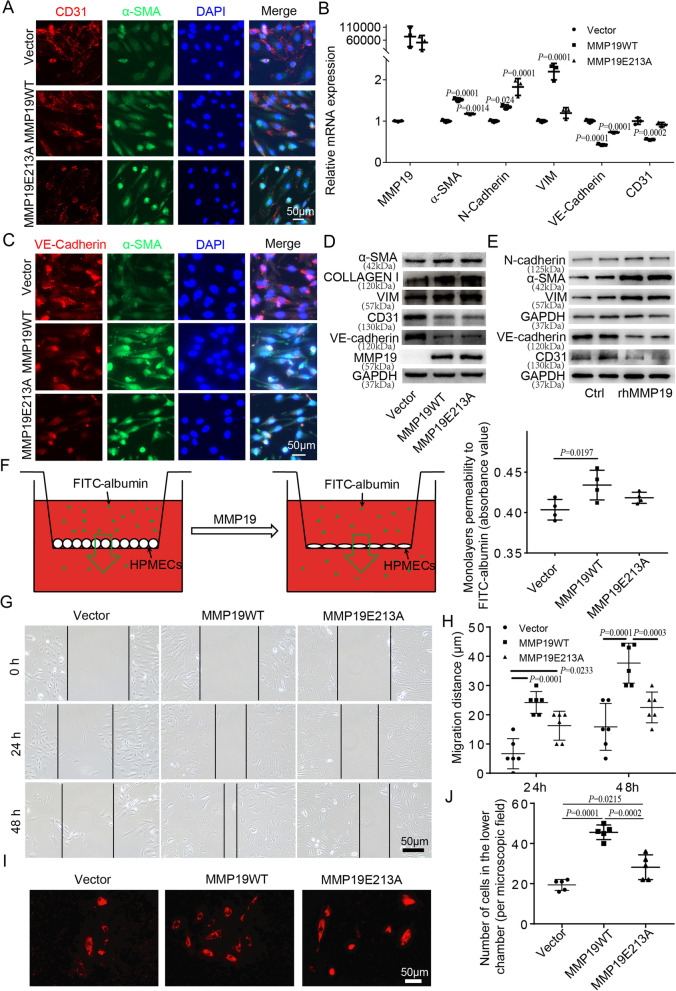
MMP19 promotes E(nd)MT, migration and permeability of HPMECs. **A** Immunofluorescence analysis for CD31 (red) and α-SMA (green) in HPMECs treated as indicated. **B** Immunofluorescence analysis for VE-Cadherin (red) and α-SMA (green) in HPMECs treated as indicated. **C** qRT-PCR was performed for markers of E(nd)MT in HPMECs 6 h after transient transfection with full length MMP19 and MMP19E213A cDNA (CDH2 = N-cadherin; VIM = vimentin). **D** Immunoblot for markers of E(nd)MT in HPMECs 24 h after transient transfection with MMP19WT and MMP19E213A cDNA. **E** Immunoblot for markers of E(nd)MT in HPMECs 24 h after rhMMP19 treatment. **F** Assay of permeability of HPMECs monolayers following transfection of MMP19WT, and MMP19E213A cDNA to FITC-albumin. **G** Scratch assay was performed on HPMECs following transfection of MMP19WT and MMP19E213A cDNA. **H** Quantification of the cell migration distance. **I** Transwell assay was used to measure HPMECs migration. **J** Quantification of the number of cells that migrated to lower chambers

To investigate the role of MMP19 in E(nd)MT in vitro, MMP19WT or MMP19E213A cDNA was transfected into HPMECs. Microscopy revealed that MMP19WT- and MMP19E213A cDNA-transfected HPMECs exhibited morphology changes associated with mesenchymal cells, and colocalization analysis showed that CD31 and VE-cadherin colocalized with a-SMA (Fig. 3A, B). In addition, MMP19WT- and MMP19E213A cDNA-transfected HPMECs showed decreased expression of CD31 and VE-cadherin and increased expression of α-SMA, vimentin, and collagen I compared to those in the control group (Fig. 3C, D). Additionally, HPMECs treated with rhMMP19 showed a decreased expression of CD31 and VE-cadherin and increased expression of α-SMA, vimentin, and N-cadherin compared to those in the control group (Fig. 3E).

The role of MMP19 in HPMECs permeability was determined by measuring FITC-albumin fluorescence intensity in the media in the lower chambers, and we found that MMP19WT cDNA-transfected HPMEC monolayers showed an increase in fluorescence intensity compared to MMP19E13A cDNA-transfected HPMECs and the control group (Fig. 3F), suggesting increased barrier permeability in MMP19WT cDNA-transfected HPMECs.

The role of MMP19 in the migration of HPMECs was examined by scratch assays. MMP19WT cDNA-transfected cells showed increased migration compared with the control group or MMP19E213A cDNA-transfected cells (Fig. 3G, H). The result were further confirmed by a transwell migration assay. As expected, cells in the lower transwell chamber in the MMP19WT cDNA-transfected group significantly outnumbered those in the MMP19E213A cDNA-transfected group and the control group (Fig. 3I, J), suggesting that MMP19 promoted the migration of HPMECs.

### MMP19 promotes E(nd)MT and vascular permeability in vivo

**Fig. 4:**
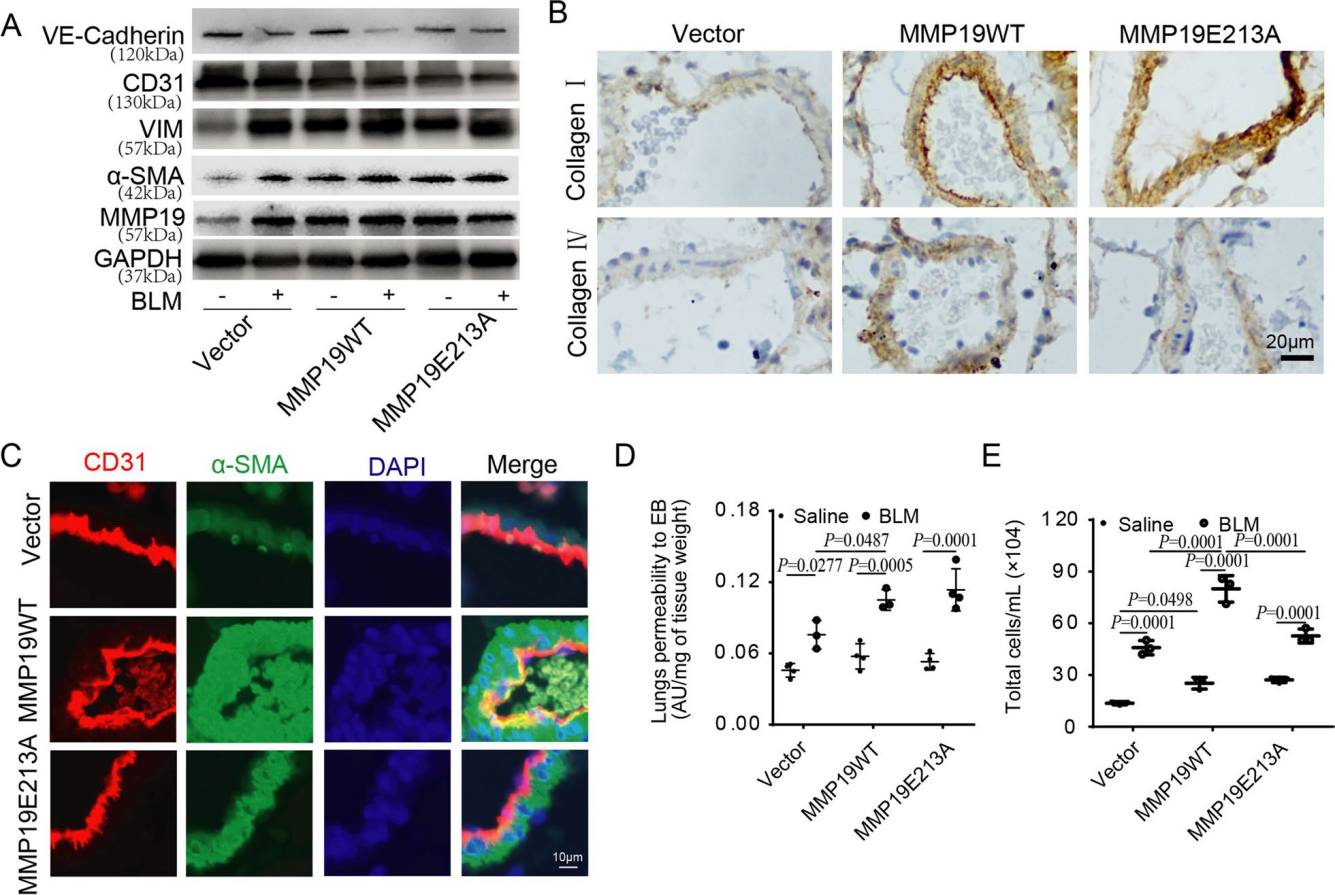
MMP19 promotes E(nd)MT and vascular permeability. **A** Western blot was performed for markers of E(nd)MT in lung homogenates of MMP19WT- and MMP19E213A-AAV-infected mice treated with saline control or BLM. **B** Immunohistochemical staining of lung sections for Collagen I and Collagen IV. **C** Immunostaining was performed to localize CD31 (red) and α-SMA (green) in endothelial cells in mice. n = 3 in vector and MMP19-AAV-infected mice treated with bleomycin, and n = 4 in other groups. **D** Assay of permeability of vascular permeability to EB. **E** Total cells in bronchoalveolar lavage (BAL) fluid. n = 3 in all groups

To investigate the contribution of MMP19 to E(nd)MT in vivo, the expression of endothelial/mesenchymal markers in lung tissues was measured by western blot, IHC analysis and IF analysis. We found augmented expression of vimentin and α-SMA, and decreased expression of CD31 and VE-cadherin in the lung tissues of MMP19WT- and MMP19E213A-AAV-infected mouse (Fig. 4A). IHC analysis showed that MMP19WT and MMP19E213A induced the expression of Collagen I and Collagen IV in endothelial cells (Fig. 4B) (Additional file 2: Fig. S2). In addition, confocal imaging showed that α-SMA colocalized with CD31 in the endothelial cells of MMP19WT- and MMP19E213A-AAV-infected mouse, and the expression of α-SMA was significantly increased in these two groups compared with the control group, while the expression of CD31 was significantly decreased (Fig. 4C). Gene expression profiles from GEO datasets (GSE181508) indicated that Mmp19 was significantly increased in the lung endothelial cells of BLM-treated mice compared to normal controls, and Mmmp19 expression was positively correlated with vimentin expression, but negatively correlated with VE-cadherin expression (Additional file 3: Fig. S3). Therefore, MMP19 promoted E(nd)MT in vivo.

Blood vessel endothelial permeability was assessed by measuring the total cells in BAL fluid and Evans blue (EB) dye that infiltration into lung tissues after tail-vein injection. The results showed increased deposition of EB in MMP19WT- and MMP19E213A-AAV-infected mouse lungs after BLM challenge compared to the control group (Fig. 4D). In addition, the total cell count in BAL fluid was significantly higher in these two groups (Fig. 4E), indicating an increase in pulmonary vascular permeability in MMP19WT and MMP19E213A-AAV-infected mice after BLM challenge.

### MMP19 induces E(nd)MT is associated with ET1

**Fig. 5:**
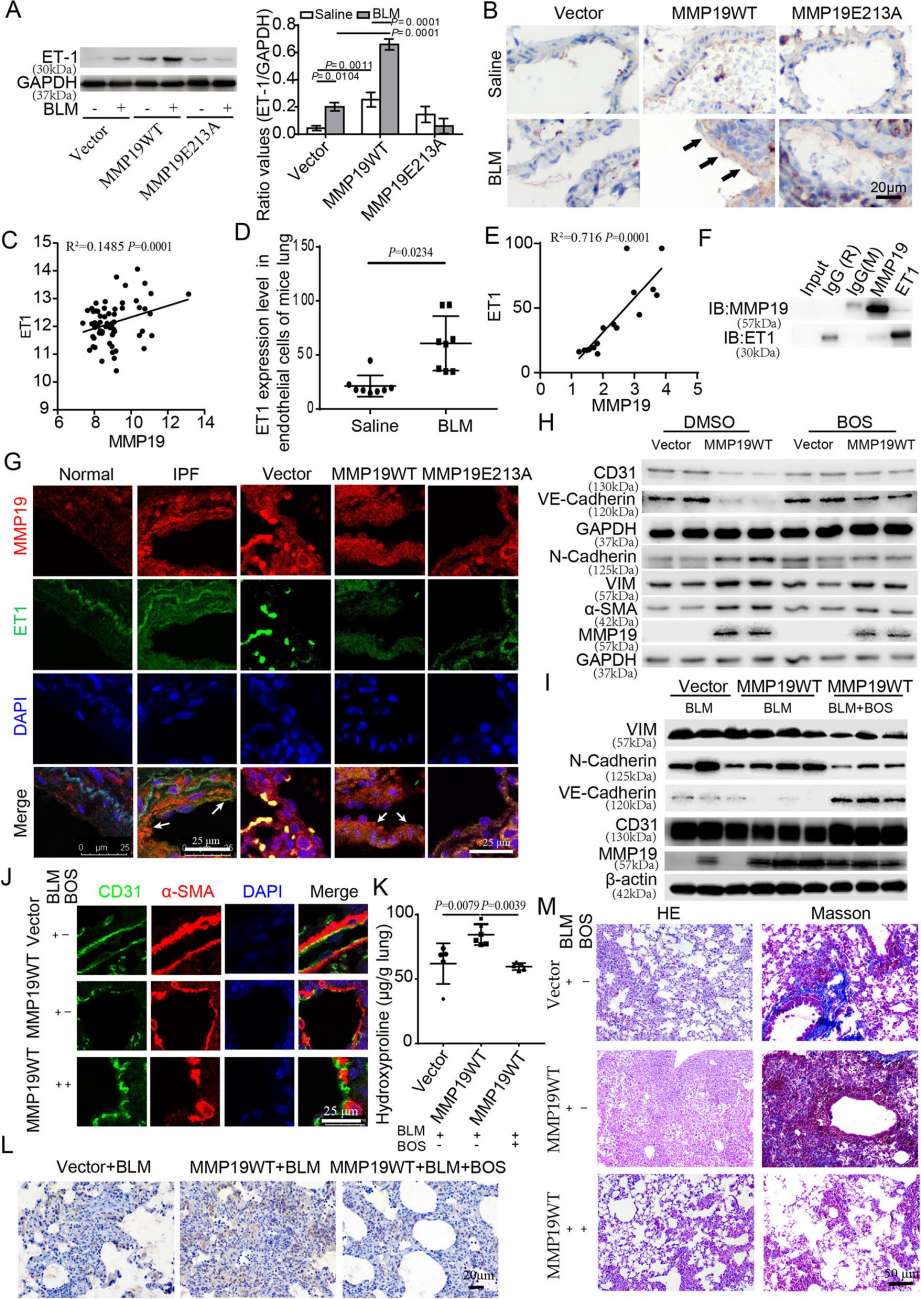
MMP19 induces E(nd)MT is associated with ET1. **A** Western blot was performed for ET1 in lung homogenates that MMP19WT- and MMP19E213A- AAV-infected mice treated with saline control or BLM. **B** Micrographs of immunohistochemical staining of lung sections for ET1. **C** Expression relevance between MMP19 and ET1 in IPF lung tissues. **D** ET1 expression level in lung endothelial cells of mice treated with saline and BLM. **E** Expression relevance between MMP19 and ET1 in lung endothelial cells of mice treated with saline and BLM. **F** MMP19 and ET-1 were assessed by immunoprecipitation in HPMECs (M = mouse, R = rabbit). **G** Immunostaining was performed to localize MMP19 (red) and ET-1 (green) in endothelial cells in IPF and mice lung tissues. **H** HPMECs were pretreated with BOS (10 µM) or DMSO for 24 h, then transient transfection with full-length MMP19 cDNA, and CD31, VE-Cadherin, N-Cadherin, Vimentin and α-SMA were analyzed by Western blotting. **I** MMP19-AAV-infected mice were pretreated with BLM, then daily gastric infusion of BOS, and CD31, VE-Cadherin, N-Cadherin and Vimentin were analyzed by Western blotting. **J** Immunofluorescence analysis for CD31 (green) and α-SMA (red) in endothelial cells of MMP19WT-AAV-infected mice treated with BLM and BOS. **K** Lung hydroxyproline levels in MMP19WT-AAV-infected mice treated with BLM and BOS. n = 5 in all groups. **L** Micrographs of immunohistochemical staining of lung sections for EMR1. **M** Representative micrographs of H&E staining and Masson’s trichrome of lung sections

ET1 is an indicator of endothelial cell injury and dysfunction. We found that ET-1 expression was significantly upregulated in the lung tissue of MMP19WT- and MMP19E213A-AAV-infected mice after BLM instillation compared to that in the control groups (Fig. 5A). In addition, IHC analysis showed that ET1 expression in endothelial cells was significantly increased in MMP19WT- and MMP19E213A-AAV-infected mice after BLM instillation compared with the control groups (Fig. 5B). The Gene GEO datasets (GSE47460) showed that the expression level of ET1 in IPF was positively correlated with MMP19 expression (Fig. 5C). In addition, the expression level of ET1 in the endothelial cells of BLM-treated mice was significantly upregulated compared to that in the control group (GSE181508) (Fig. 5D), and its expression was positively correlated with MMP19 (Fig. 5E). These results indicated that MMP19 was closely related to ET1 in lung fibrosis.

To investigate the interaction between MMP19 and ET1, we performed a Co-IP assay in HPMECs, and found that MMP19 coimmunoprecipitated with ET1 (Fig. 5F). In addition, confocal microscopy confirmed that ET1 colocalized with MMP19, and ET1-positive staining was significantly stronger in the endothelial cells of MMP19WT- and MMP19E213A-AAV-infected mice and IPF lung tissues than in corresponding control group lung tissues (Fig. 5G).

To further determine the role of ET1 in MMP19-induceded E(nd)MT, we evaluated the effect of BOS, which is an antagonist of the ET1 receptor, on MMP19-induced E(nd)MT and pulmonary fibrosis severity in the MMP19WT-AAV-infected mice after BLM instillation. We found that BOS significantly inhibited MMP19 induced expression of N-cadherin and vimentin and downregulation of CD31 and VE-cadherin in vitro and in vivo (Fig. 5H–J). In addition, BOS significantly reduced the number of F4/80^+^ macrophages in MMP19WT-AAV-infected mice lung tissues (Fig. 5L), and alleviated MMP19-induced lung fibrosis, as evidenced by decreased hydroxyproline levels and collagen deposition in BLM challenged mouse lungs (Fig. 5K, M). Thus, BOS inhibited MMP19-induced E(nd)MT and alleviated MMP19 mediated exacerbation of BLM-induced pulmonary fibrosis.

### MMP19 induces monocyte adhesion and infiltration is associated with the SDF1/CXCR4 axis

**Fig. 6:**
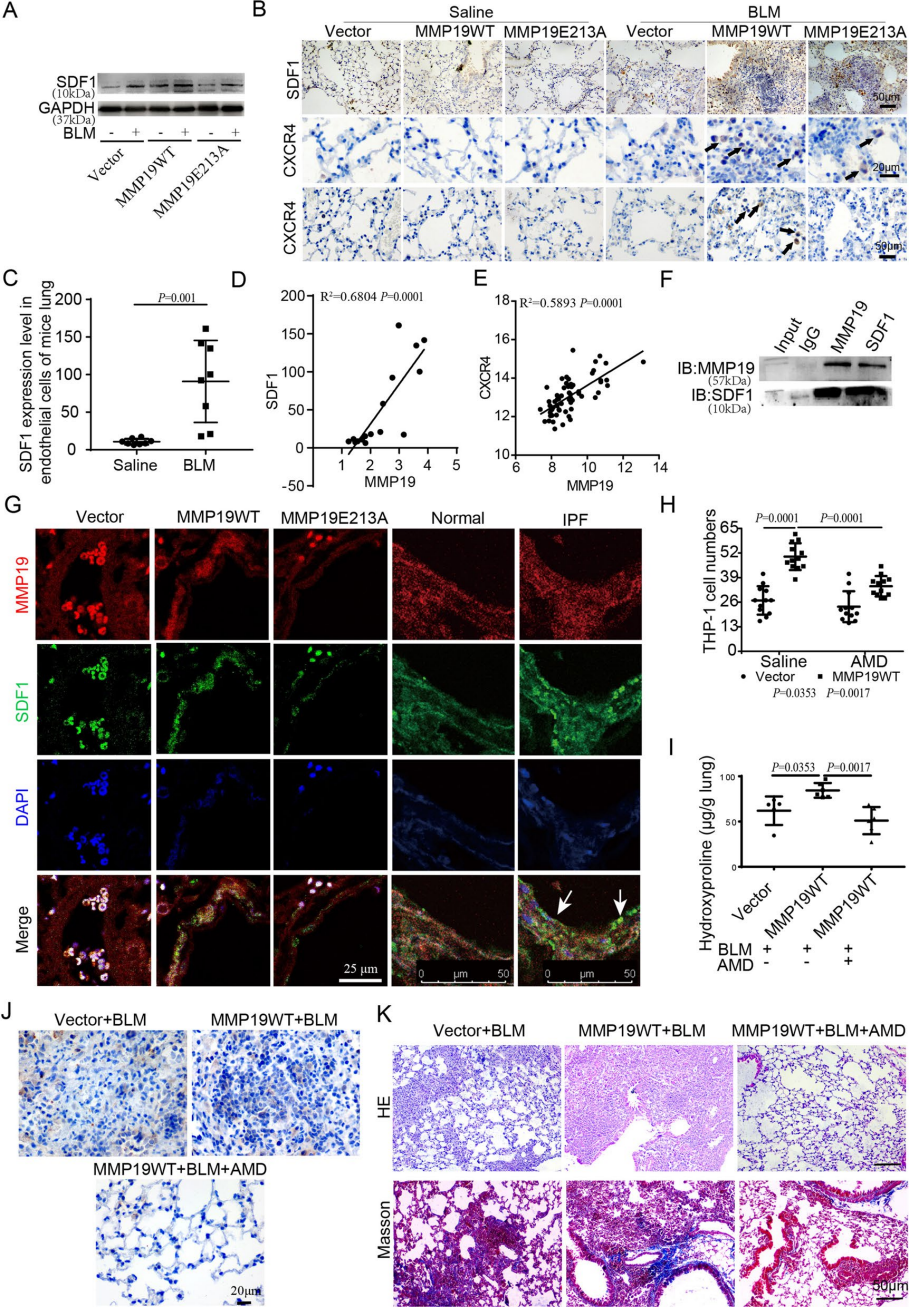
MMP19 induces monocyte infiltration is associated with the SDF1/CXCR4 axis. **A** Western blot was performed for SDF1 in lung homogenates that MMP19WT and MMP19E213A-AAV-infected mice treated with saline control or BLM. **B** Representative micrographs of immunohistochemical staining for SDF1 and CXCR4 in lung sections (up and middle), and CXCR4 in blood vessels (bottom). **C** SDF1 expression level in lung endothelial cells of mice treated with saline and BLM. **D** Expression relevance between MMP19 and SDF1 in lung endothelial cells of mice treated with saline and BLM. **E** Expression relevance between MMP19 and CXCR4 in IPF lung tissues. **F** MMP19 and SDF1 were assessed by immunoprecipitation in HPMECs. **G** Immunofluorescence was performed to localize MMP19 (red) and SDF-1 (green) in endothelial cells in mice and IPF lung tissues. **H** THP-1 cell adhesion to HPMECs was assessed using an inverted fluorescence microscope. **I** Lung hydroxyproline levels in MMP19WT-AAV-infected mice treated with BLM and AMD3100. n = 5 in all groups. **J** Micrographs of immunohistochemical staining of lung sections for EMR1. **K** Representative micrographs of H&E staining and Masson’s trichrome of lung sections

C–X–C motif chemokine ligand 12 (CXCL12, SDF1) has been reported to regulate monocyte adhesion. Western blot and IHC analysis showed that SDF-1 expression was significantly upregulated in lung tissue of MMP19WT- and MMP19E213A-AAV- infected mice after BLM instillation compared to the control (Fig. 6A, B). IHC analysis further showed that positive CXCR4 staining was significantly increased in these two groups (Fig. 6B). Moreover, CXCR4^+^ cells in the blood vessels in the lungs of MMP19WT- and MMP19E213A-AAV-infected mice after BLM instillation were significantly increased compared to those in the control groups (Fig. 6B). GEO datasets (GSE181508 and GSE47460) were analyzed, and we found that the expression level of SDF1 in the endothelial cells of mice with BLM-induced fibrosis was significantly upregulated compared to that in the control group (Fig. 6C), and SDF1 expression in IPF lungs and the endothelial cells of BLM-induced fibrotic mouse lungs was positively correlated with MMP19 expression (Fig. 6D). Moreover, the expression of the receptor CXCR4 in IPF was positively correlated with MMP19 expression (Fig. 6E). These results indicated that MMP19 was closely related to SDF1 in lung fibrosis.

To investigate the interaction between MMP19 and SDF-1, we performed a Co-IP assay in HPMECs, and found that MMP19 coimmunoprecipitated with SDF-1 (Fig. 6F). In addition, confocal analysis confirmed that SDF-1 colocalized with MMP19, and SDF-1-positive staining was significantly stronger in the endothelial cells of MMP19WT- and MMP19E213A-AAV-infected mice and IPF lungs compared to that in the corresponding control lung (Fig. 6G). Furthermore, we evaluated the effect of AMD3100, a small molecule inhibitor of CXCR4, on MMP19-induced monocyte adhesion and infiltration. We found that AMD3100 significantly inhibited MMP19 induced monocyte adhesion to HPMECs and CXCR4^+^ monocyte accumulation in lung tissues (Fig. 6H, I), and alleviated the MMP19-induced increase in hydroxyproline levels and accumulation of ECM and collagen in BLM-induced pulmonary fibrosis (Fig. 6J, K). Thus, AMD3100 alleviated the MMP19-induced exacerbation of monocyte adhesion, infiltration and BLM-induced pulmonary fibrosis.

## Discussion

Myofibroblast activation and excessive ECM deposition are known pathological features of end-stage IPF. Our previous study indicated that Mmp19^−/−^ mice showed a significantly increased susceptibility to the BLM-induced fibrosis in the lung compared with WT mice [8]. During this process, excessive ECM was deposited due to the absence of MMP19. However, contrary to our initial hypothesis, increased expression of MMP19 could promote the degradation of ECM and inhibit pulmonary fibrosis. In this study, we found that MMP19WT- or MMP19E213A-AAV-infected mice exhibited more severe pulmonary fibrosis after BLM instillation than control mice. Therefore, we hypothesized that forced expression of MMP19 increased the activation and number of myofibroblasts in lung tissue, and aggravated BLM-induced pulmonary fibrosis by activating its effector cells. MMP19 was significantly increased in the endothelial cells of IPF and BLM-induced fibrotic mouse lung tissues. The expression of vimentin in the endothelial cells of BLM-induced fibrotic mouse lung tissues was positively correlated with that of MMP19, while the expression of VE-cadherin was negatively correlated with that of MMP19. These results suggested a crucial role for MMP19 in E(nd)MT and pulmonary fibrosis.

Studies have demonstrated that vessel density in the fibrotic areas of IPF lung tissues was lower than in the nonfibrotic regions [16, 17], and the intimal layer of the artery is damaged with an increase in ECM deposition [18]. In radiation-induced pulmonary fibrosis, E(nd)MT occurred principally in large vessels and appeared before the development of EMT in alveoli [19], indicating that E(nd)MT was an important inducer of pulmonary fibrosis. In this study, the role of MMP19 in regulating E(nd)MT was assessed in MMP19-transfected HPMECs and MMP19-AAV-infected mice, and we found that MMP19 promoted E(nd)MT, which was consistent with the finding that LIPUS alleviated the migration of E(nd)MT-derived mesenchymal-like cells by reducing ECM deposition is associated with MMP proteolytic activity [20]. During E(nd)MT, endothelial cells lose their marker molecules and obtain mesenchymal cell markers, resulting in an obvious honeycomb structure in the vascular endothelial layer [21], thereby destroying the stability of endothelial connections and increasing vascular permeability [22–24]. In this study, we also found that the FITC-albumin fluorescence intensity in the media in the lower chambers of MMP19WT-transfected HPMEC monolayers and the EB intensity in MMP19-WT-AAV-infected mice after BLM instillation were significantly increased compared with those in the corresponding control groups. This finding indicated that MMP19 increased the permeability of the endothelial barrier by inducing E(nd)MT. Our observation that MMP19WT- and MMP19E213A-AAV-infected mice showed exaggerated monocyte accumulation in BAL fliud (Additional file 4: Fig. S4) was consistent with these observations. Interestingly, we observed that MMP19-AAV-infected mouse lung tissues showed a faster settling velocity in paraformaldehyde than the control tissue (Additional file 5: Fig. S5), which further indicated that MMP19 increased vascular permeability. Studies have shown that E(nd)MT increase the migration of endothelial cells in vitro, and the migration of HMEC-1 cells undergoing E(nd)MT is dependent on ECM degradation and invadosome formation associated with MMP-2 proteolytic activity [25]. In this study, we found that MMP19 promoted the migration of endothelial cells in transwell and scratch assays. Studies have shown that E(nd)MT is associated with increased proliferation of ECs [26–28]. Additionally, we found that MMP19 promoted the proliferation of HPMECs (Additional file 6: Fig. S6). Taken together, these results demonstrated that upregulation of MMP19 in endothelial cells promoted E(nd)MT and promoted the migration and proliferation of endothelial cell-derived fibroblasts by inducing E(nd)MT, and finally exacerbating BLM-induced pulmonary fibrosis.

The E(nd)MT caused by ECM alterations was closely related to the enzymatic activity of MMPs. To determine the role of the enzymatic functions of MMP19 in E(nd)MT, we used a catalytic site mutant. A mutation in the zinc-binding motif of MMP19 from glutamate (E) to alanine (A) (MMP19E213A) causes a loss of function in protein degradation [29]. Interestingly, after BLM challenge, MMP19WT-AAV-infected mice showed more severe vascular remodeling and fibrosis than MMP19E213A-AAV- infected mice, while MMP19WT and MMP19E213A induced E(nd)MT in HPMECs. Sadowski et al. showed that MMP19WT but not MMP19E213A induced ECM degradation and the migration of epithelial cells, and participated in the early stages of squamous cell cancer (SCC) invasion [30]. Consisting with their findings, our data suggested that MMP19 but not MMP19E213A promoted the migration and permeability of HPMECs. After BLM instillation, MMP19-AAV-infected mice showed more cell numbers in BAL fliud and more monocyte infiltration in the lungs than MMP19E213A-AAV-infected mice. Therefore, the enzymatic activity of MMP19 was crucial for enhancing the permeability of endothelial cells, promoting monocyte infiltration and aggravating BLM-induced pulmonary fibrosis.

ET1 is mainly produced by injured endothelial cells, and has been reported to stimulate the synthesis of collagen and promote E(nd)MT, which indicates that ET1 enhances fibrosis by inducing E(nd)MT [31–33]. In the uteroplacental perfusion pressure (RUPP) rat model, the activation of MMPs in endothelial cells increased ET1 release and led to endothelial dysfunction in the maternal system [34]. At sites of tissue injury and inflammation, MMPs increased the expression of ET1 and promoted leukocyte-endothelial cell adhesion and neutrophil trafficking into inflamed tissues. Taken together, these findings indicated the synergistic association between at least some MMPs and ET-1 in endothelial cell stability and adhesion. In this study, we found that MMP19 significantly increased the expression of ET1 in lung endothelial cells and lung tissues. Co-IP analysis showed that MMP19 was associated with ET1 in HPMECs. We also found that the expression of ET1 in BLM-induced MMP19-AAV-infected mice and IPF lung was significantly higher than that in control lung tissues, which further indicated that MMP19-induced E(nd)MT and pulmonary fibrosis were associated with ET1. To further explore the role of ET1 in MMP19-induced E(nd)MT, BOS was used to treat MMP19 cDNA-transfected HPMECs and MMP19-AAV-infected mice followed by BLM challenge. We found that BOS inhibited MMP19-induced E(nd)MT and F4/80^+^ macrophage accumulation in lung tissues, and alleviated MMP19 mediated exacerbation of BLM-induced pulmonary fibrosis. This result was consistent with the finding that BOS prevented the increase in lung vascular permeability induced by moderate hypoxia and oleic acid [35, 36], and reduced BLM-induced pulmonary fibrosis [37, 38]. In this regard, MMP19-induced E(nd)MT was associated with ET-1.

The expression of SDF1 and CXCR4 was increased in lung tissues from Day 3 and until Day 21 after BLM treatment [39], downregulation of SDF1/CXCR4 axis could inhibit fibrosis and thrombosis in the presence of tetramethylpyrazine [40]. Recent studies showed that CXCR4^+^ macrophage cells were increased in the lung tissue of IPF [41], indicating that CXCR4^+^ macrophage infiltration in lung tissues plays an important role in pulmonary fibrosis. In this study, increased expression of SDF-1 and CXCR4 was observed in MMP19WT-AAV-infected mice after BLM instillation and in IPF lung tissues. It has been reported that MMP-2 in tumor cells increases the expression of SDF1, leading to increased stem cell tropism toward tumor cells via the SDF1/CXCR4 axis [42]. MMP9 and SDF1 perform a synergistic partnership in facilitating transmigration of monocytes into the injured spinal cord [43]. Taken together, these findings indicate a close association between at least some MMPs and SDF1 in regulating macrophage adhesion and infiltration. In this study, we found that MMP19 was associated with SDF1 in HPMECs by Co-IP. Additionally, we found that SDF1 expression was significantly upregulated in lung endothelial cells and the lung tissues of MMP19WT- and MMP19E213A-AAV-infected mice after BLM instillation, and CXCR4^+^ cells were significantly increased in the lung tissues and blood vessels of MMP19-AAV-infected mice. These findings indicated that MMP19 increased the expression of SDF1 in lung endothelial cells and lung tissues, recruited CXCR4^+^ immune cells, facilitated their infiltration in the lung, and aggravated BLM-induced pulmonary fibrosis. To further elucidate the role of the SDF1/CXCR4 axis in MMP19 induced monocyte adhesion and infiltration, AMD3100 was used to treat MMP19 cDNA-transfected HPMECs and MMP19-AAV-injected mice followed by BLM challenge. We found that AMD3100 inhibited MMP19-induced monocyte adhesion to HPMECs and CXCR4^+^ monocyte accumulation in lung tissues, and alleviated MMP19-induced exacerbation of BLM-induced pulmonary fibrosis. This result was consistent with the finding that AMD3100 blocks the accumulation of T cells and pulmonary fibrosis in Twist1-null mice [44]. Therefore, MMP19 induced monocyte adhesion and infiltration were associated with the SDF1/CXCR4 axis.

Crosstalk between epithelial cells and endothelial cells might play an important role in maintaining airway epithelial structure. EMT in AE cells may be indirectly caused by E(nd)MT and hypoxia, in addition to the direct damage to AE cells caused by irradiation in RIPF [19]. In radiation proctitis, endothelial cell-specific knockout of Hey2 may protect the endothelial and the epithelial cell compartment from radiation damage [45]. These studies suggested that E(nd)MT was one of the inducers of epithelial cell damage. Ding et al. showed that unilateral pneumonectomy stimulates PCECs to produce angiocrine growth factors that induce the proliferation of epithelial progenitor cells that support alveologenesis [46]. We found that SPC^+^ cell numbers in BLM-challenged MMP19-AAV- and MMP19E213A-AAV-infected mice were significantly reduced compared with the control groups (Additional file 7: Fig. S7A). BOS inhibited E(nd)MT, and increased SPC^+^ cell number in BLM-challenged MMP19-AAV-infected mice (Additional file 7: Fig. S7B). Our previous study showed that silencing MMP19 significantly reduced epithelial cell migration and wound healing, indicating that the expression of MMP19 in epithelial cells is necessary for the recovery of injured epithelial cells and ameliorating BLM-induced pulmonary fibrosis [8]. It seems that MMP19 plays different roles in endothelial cells and epithelial cells in BLM-induced pulmonary fibrosis. In detail, deletion of MMP19 in mouse lungs accelerated the deposition of collagen, inhibited the repair of injured epithelial cells, and aggravated BLM-induced pulmonary fibrosis. The overexpression of MMP19 in endothelial cells promoted E(nd)MT, stimulated epithelial cell damage, and activated fibroblasts. Additional experiments will be required to address this issue.

## Conclusion

**Fig. 7:**
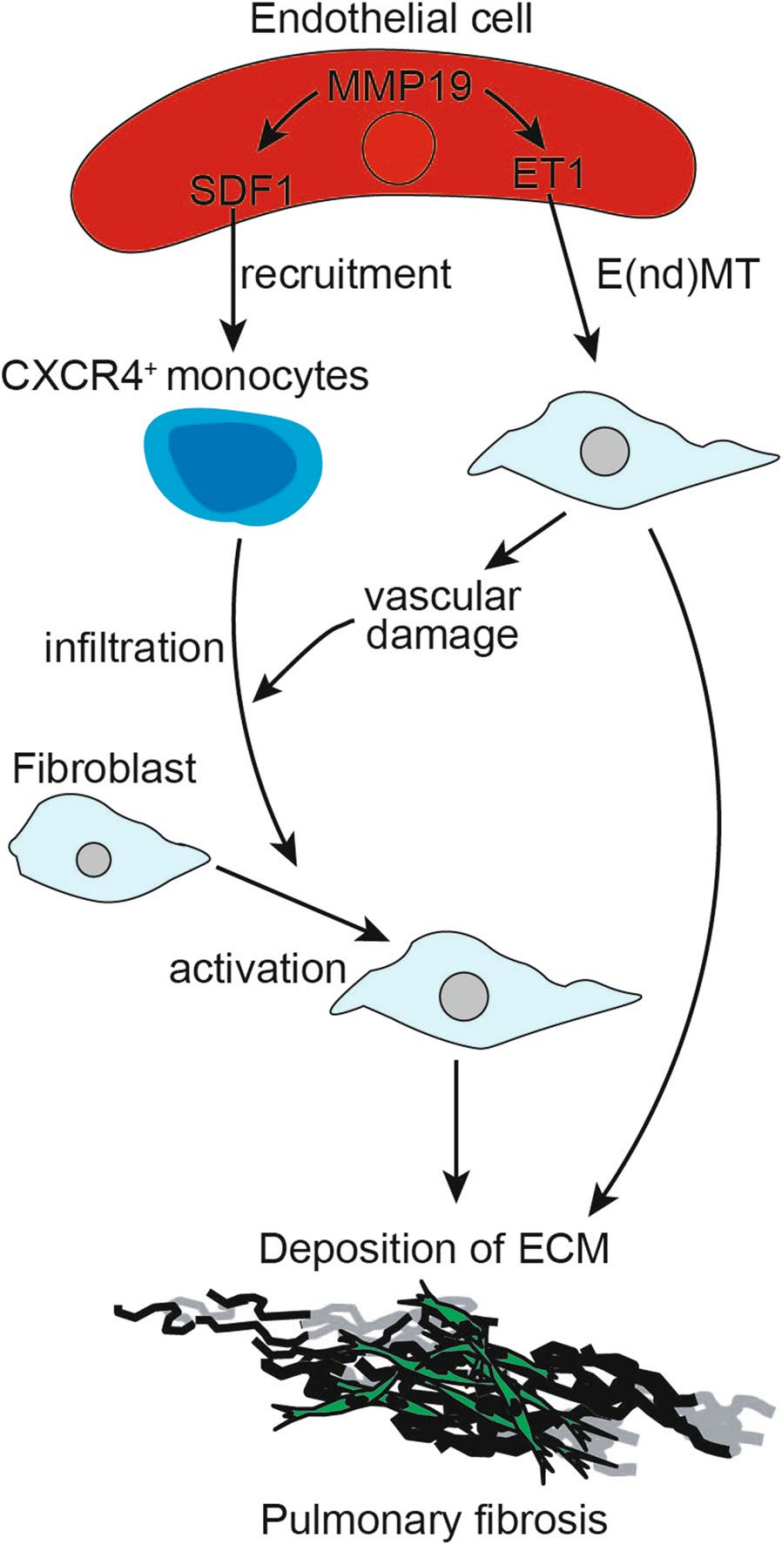
Schematic model of MMP19 involvement in pathophysiological processes of E(nd)MT and pulmonary fibrosis. Overexpression of MMP19 in endothelial cells promoted E(nd)MT of endothelial cells and vascular permeability and led to more monocytes infiltration into bronchoalveolar lavage and tissue lumen through the induction of ET1 and SDF1 in endothelial cells, further facilitating fibrotic microenvironment with abundant of growth factors, cytokines, chemokines, and aggravated pulmonary fibrosis

In summary, in IPF and BLM-induced fibrosis in mice, endothelial cell-derived MMP19 promoted the expression of ET1, which in turn aggravated E(nd)MT and vascular permeability. MMP19 also promoted the expression of SDF1 in endothelial cells, which in turn promoted the adhesion of CXCR4^+^ monocytes to endothelial cells and the infiltration of monocytes into lung tissue, ultimately aggravating the development of IPF (Fig. 7). MMP19 plays an important role in microvascular endothelial cell injury, activation and remodeling, and pulmonary fibrosis and could be a promising therapeutic target for suppressing pulmonary fibrosis.

## Supplementary Information

**Additional file 1: Fig. S1**. Mouse lung fibrosis model. **A** MMP19 overexpression in mice lungs was achieved by intratracheal injection of AAV, followed by intratracheal injection of PBS or BLM at 4 days, and mice were sacrificed at 18 days. **B** In vivo imaging system was performed to detect the transfection efficiency of AAV. **C** Fluorescence microscope was performed to detect the transfection efficiency of AAV. **D** Western blot was performed to detect the transfection efficiency of AAV.

**Additional file 2:** **Fig. S2**. Densitometric analysis of the bands in Fig. 4A. **A** Densitometric analysis of the bands of MMP19 normalized with GAPDH. **B** Densitometric analysis of the bands of α-SMA normalized with GAPDH. **C** Densitometric analysis of the bands of VIM normalized with GAPDH. **D** Densitometric analysis of the bands of CD31 normalized with GAPDH. **E** Densitometric analysis of the bands of VE-cadherin normalized with GAPDH.

**Additional file 3:** **Fig. S3**. Expression profile analysis of Vimentin and VE-cadherin in endothelial cells of mice treated with Saline and BLM. **A**-**B** Expression profile of Vimentin and VE-cadherin in endothelial cells of mice treated with Saline and BLM. **C**-**D** Expression relevance between MMP19, Vimentin and VE-cadherin in endothelial cells of mice treated with Saline and BLM.

**Additional file 4:** **Fig. S4**. M2 macrophages were significantly increased MMP19WT-AAV-infected mice after BLM instillation.

**Additional file 5:** **Fig. S5**. Lung tissues fixed with 4% paraformaldehyde.

**Additional file 6:** **Fig. S6**. MMP19 promoted the proliferation of HPMECs. **A** EdU assay was used to measure HPMECs proliferation. **B** Quantification of the number of the EdU labeled cells. **C** CCK8 assay was used to measure HPMECs proliferation.

**Additional file 7:** **Fig. S7**. SPC^+^ cells in mouse lung tissues. **A** Immunofluorescence analysis for SPC in MMP19WT and MMP19E213A-AAV-infected mice treated with saline or BLM. **B** Immunofluorescence analysis for SPC in MMP19WT -AAV-infected mice treated with BOS.

## Data Availability

All data generated or analysed during this study are included in this published article [and its supplementary information files].

## Abbreviations

MMPs: Matrix metalloproteinases
ECM: Extracellular matrix
IPF: Idiopathic pulmonary fibrosis
EMT: Epithelial-mesenchymal transition
E(nd)MT: Endothelial-to-mesenchymal transition
BLM: Bleomycin
ET1: Endothelin-1
WT: Wild-type
BOS: Bosentan
Co-IP: Co-Immunoprecipitation
HE: Hematoxylin and eosin
IF: Immunofluorescence
IHC: Immunohistochemistry
