# Supplementary figures and images for: Endothelial cell-derived MMP19 promotes pulmonary fibrosis by inducing E(nd)MT and monocyte infiltration
Source: Cell Commun Signal. 2023 Mar 13;21:56. doi: 10.1186/s12964-023-01040-4 (PMC10009991; doi:10.1186/s12964-023-01040-4)

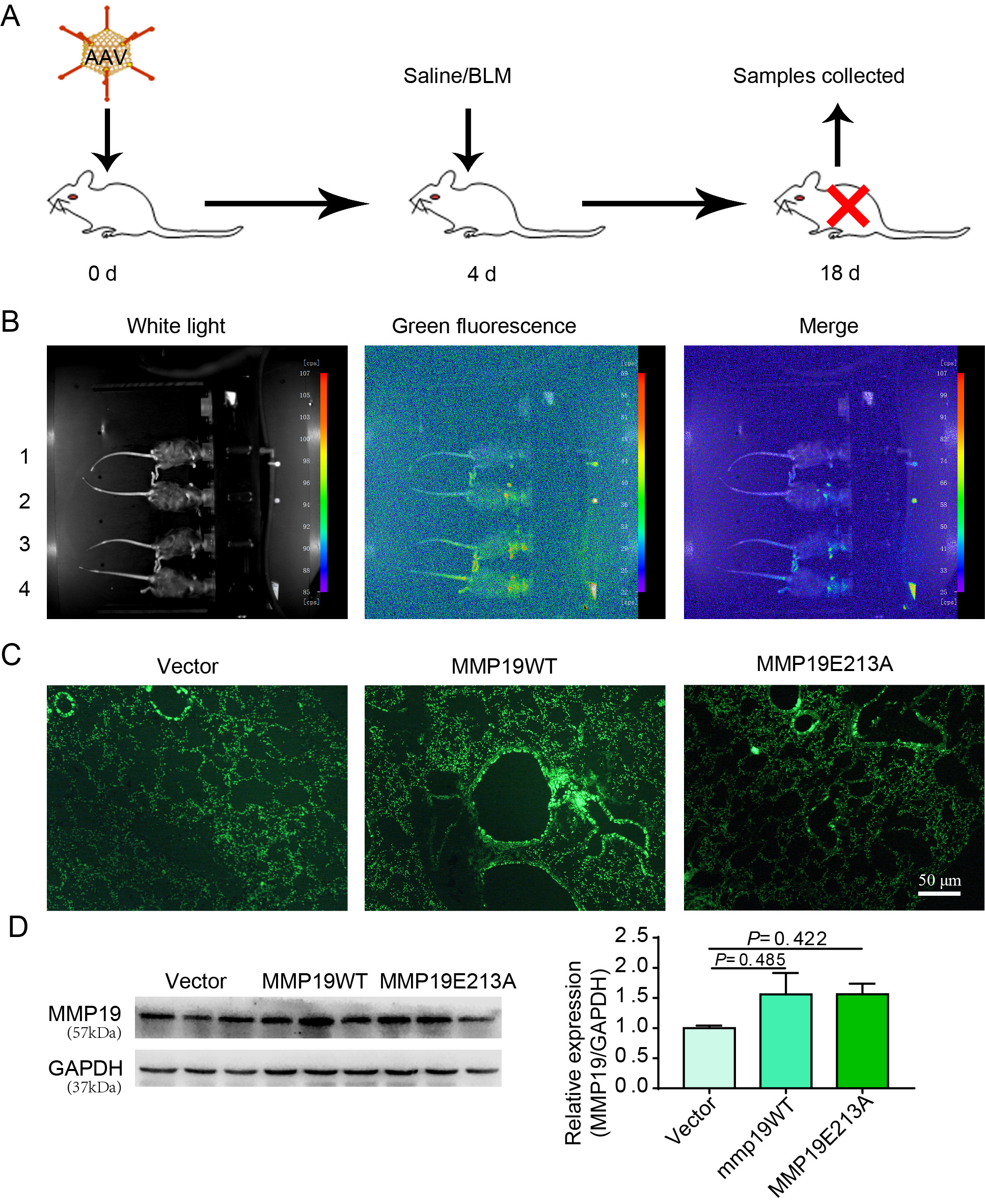

Supplement: Supplementary file 2 — Additional file 1: Fig. S1. Mouse lung fibrosis model. A MMP19 overexpression in mice lungs was achieved by intratracheal injection of AAV, followed by intratracheal injection of PBS or BLM at 4 days, and mice were sacrificed at 18 days. B In vivo imaging system was performed to detect the transfection efficiency of AAV. C Fluorescence microscope was performed to detect the transfection efficiency of AAV. D Western blot was performed to detect the transfection efficiency of AAV. [file 12964_2023_1040_MOESM2_ESM.jpg]

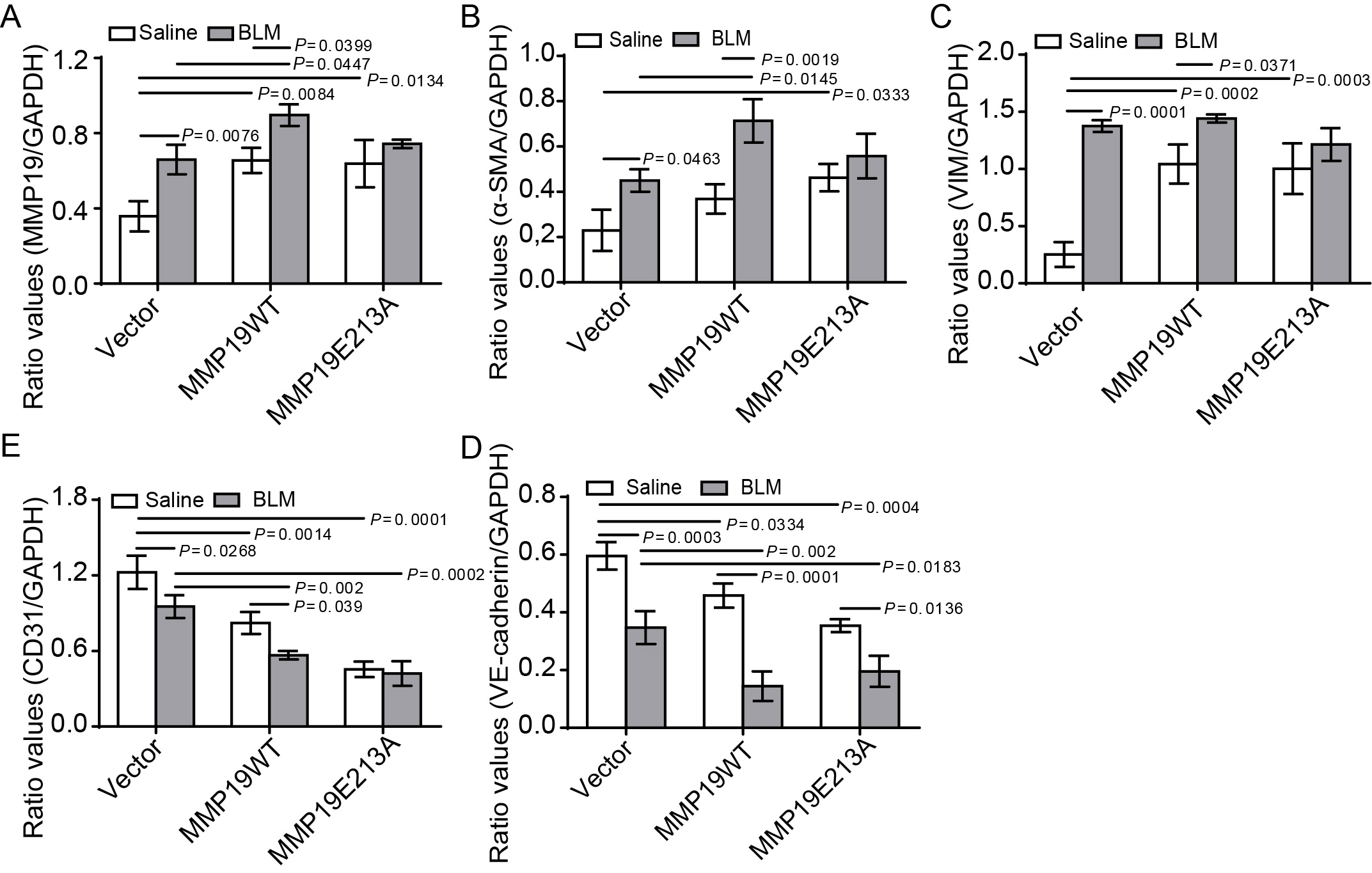

Supplement: Supplementary file 3 — Additional file 2: Fig. S2. Densitometric analysis of the bands in Fig. 4A. A Densitometric analysis of the bands of MMP19 normalized with GAPDH. B Densitometric analysis of the bands of α-SMA normalized with GAPDH. C Densitometric analysis of the bands of VIM normalized with GAPDH. D Densitometric analysis of the bands of CD31 normalized with GAPDH. E Densitometric analysis of the bands of VE-cadherin normalized with GAPDH. [file 12964_2023_1040_MOESM3_ESM.jpg]

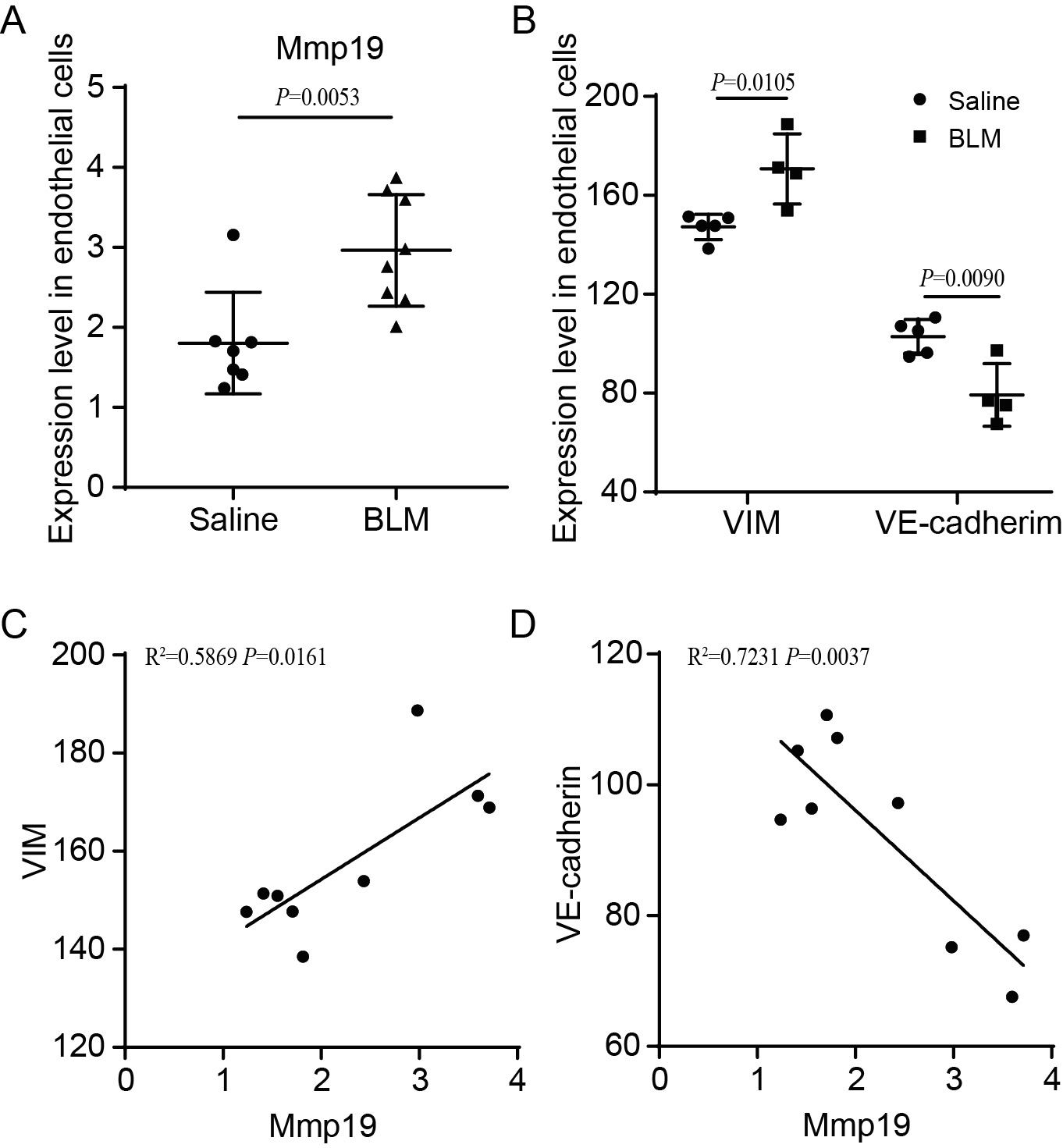

Supplement: Supplementary file 4 — Additional file 3: Fig. S3. Expression profile analysis of Vimentin and VE-cadherin in endothelial cells of mice treated with Saline and BLM. A-B Expression profile of Vimentin and VE-cadherin in endothelial cells of mice treated with Saline and BLM. C-D Expression relevance between MMP19, Vimentin and VE-cadherin in endothelial cells of mice treated with Saline and BLM. [file 12964_2023_1040_MOESM4_ESM.jpg]

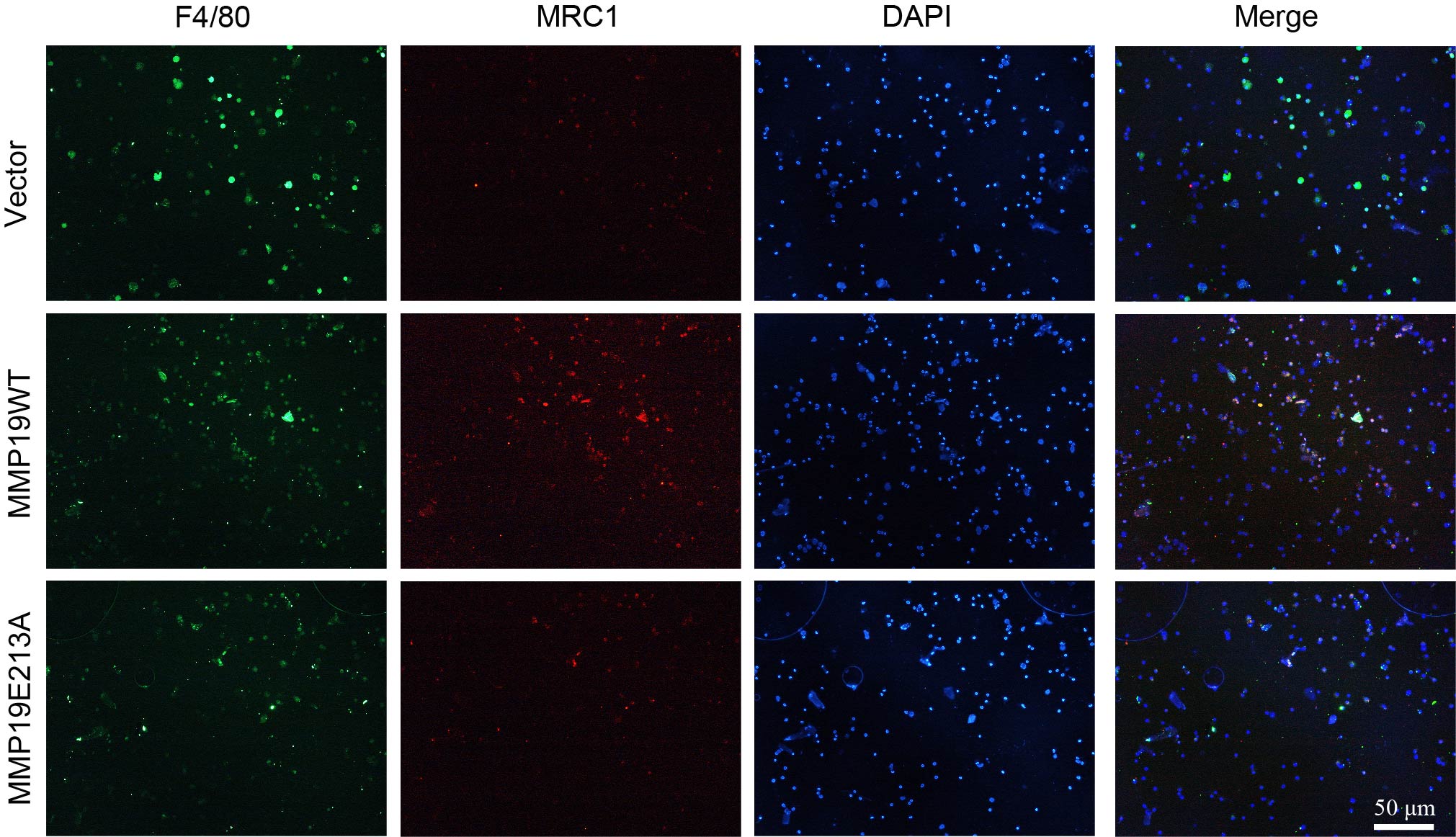

Supplement: Supplementary file 5 — Additional file 4: Fig. S4. M2 macrophages were significantly increased MMP19WT-AAV-infected mice after BLM instillation. [file 12964_2023_1040_MOESM5_ESM.jpg]

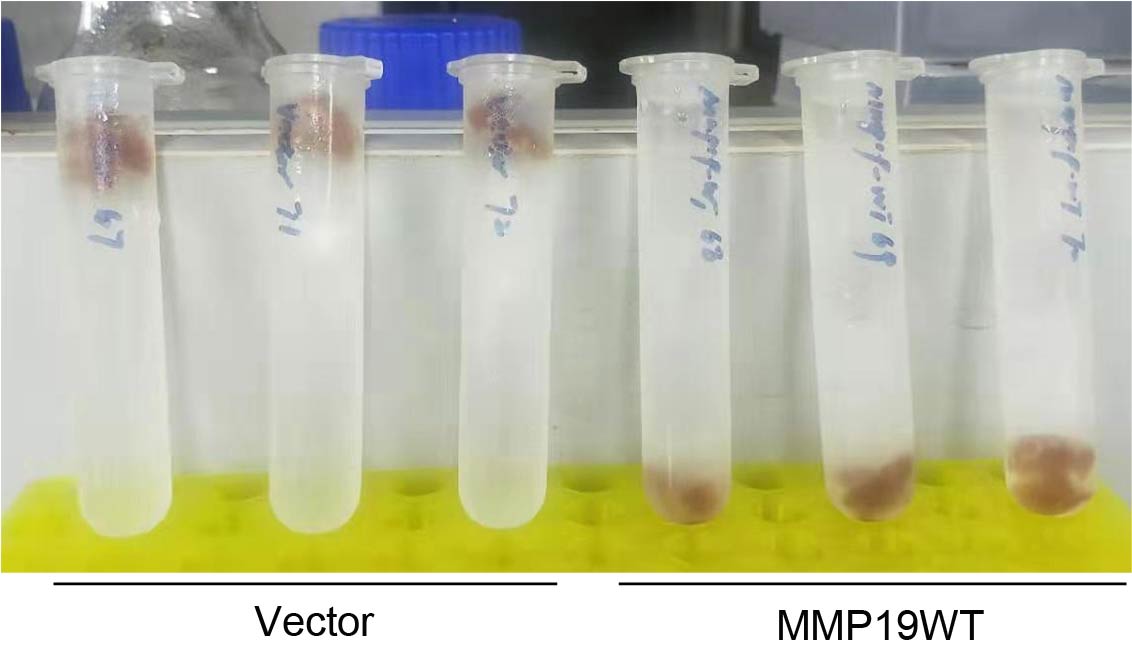

Supplement: Supplementary file 6 — Additional file 5: Fig. S5. Lung tissues fixed with 4% paraformaldehyde. [file 12964_2023_1040_MOESM6_ESM.jpg]

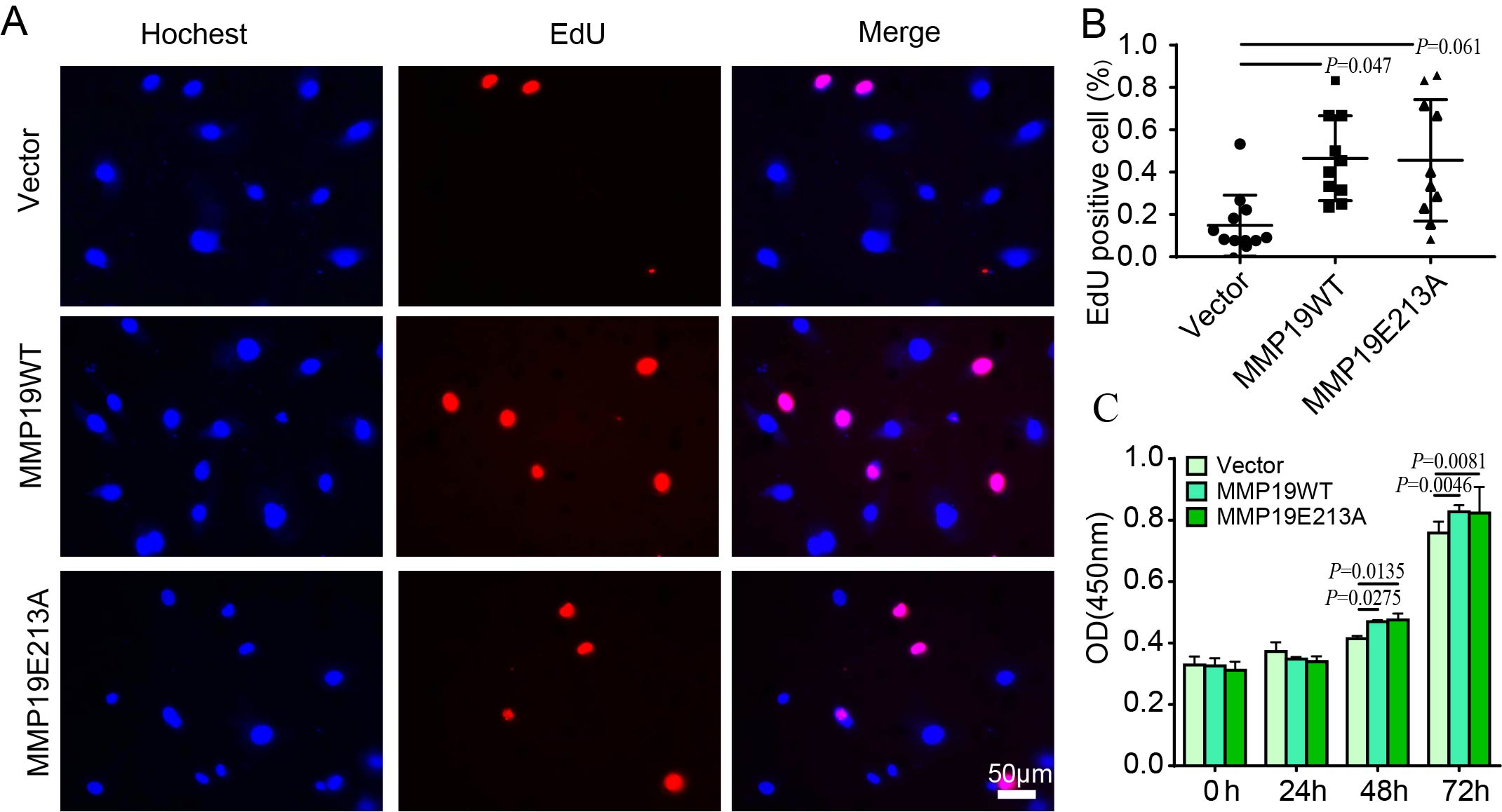

Supplement: Supplementary file 7 — Additional file 6: Fig. S6. MMP19 promoted the proliferation of HPMECs. A EdU assay was used to measure HPMECs proliferation. B Quantification of the number of the EdU labeled cells. C CCK8 assay was used to measure HPMECs proliferation. [file 12964_2023_1040_MOESM7_ESM.jpg]

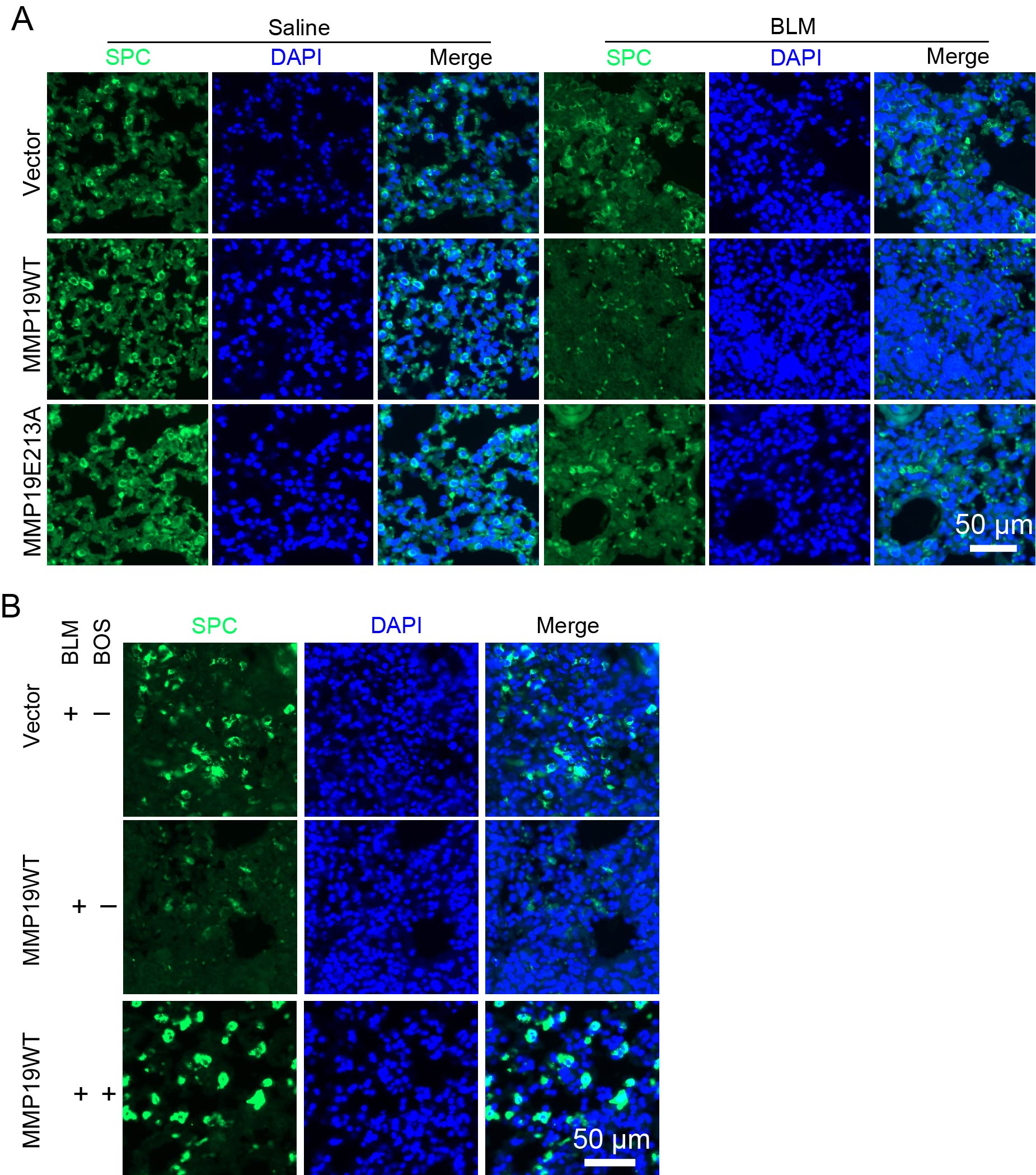

Supplement: Supplementary file 8 — Additional file 7: Fig. S7. SPC+ cells in mouse lung tissues. A Immunofluorescence analysis for SPC in MMP19WT and MMP19E213A-AAV-infected mice treated with saline or BLM. B Immunofluorescence analysis for SPC in MMP19WT -AAV-infected mice treated with BOS. [file 12964_2023_1040_MOESM8_ESM.jpg]
